# Multimodal therapy with surgery and adjuvant nivolumab for late-onset multiple liver metastases of choroidal malignant melanoma: a case report

**DOI:** 10.1186/s40792-020-00948-0

**Published:** 2020-07-31

**Authors:** Ryuta Muraki, Yoshifumi Morita, Shinya Ida, Ryo Kitajima, Satoru Furuhashi, Ryota Kiuchi, Makoto Takeda, Hirotoshi Kikuchi, Yoshihiro Hiramatsu, Takanori Sakaguchi, Akira Kasuya, Yoshihiro Hotta, Hiroya Takeuchi

**Affiliations:** 1 Department of Surgery, Hamamatsu University School of Medicine, 1-20-1 Handayama, Higashi-ku, Hamamatsu, 431-3192 Japan; 2 Department of Gastroenterological Surgery, Iwata City Hospital, Iwata, Japan; 3 Department of Perioperative Functioning Care & Support, Hamamatsu University School of Medicine, Hamamatsu, Japan; 4 Department of Dermatology, Hamamatsu University School of Medicine, Hamamatsu, Japan; 5 Department of Ophthalmology, Hamamatsu University School of Medicine, Hamamatsu, Japan

**Keywords:** Choroidal malignant melanoma, Late recurrence, Liver metastasis, Nivolumab, Adjuvant therapy

## Abstract

**Background:**

Choroidal malignant melanoma is the most common primary malignant tumor of the eye in adults. Prognosis after recurrence of this disease has been dismal because of the absence of an effective therapy. However, resection of recurrent foci and a subsequent treatment with immune-checkpoint inhibitor may improve the prognosis after recurrence of this disease. This study presents a case of late-onset liver metastases of choroidal malignant melanoma, successfully treated with hepatectomy and postoperative adjuvant nivolumab.

**Case presentation:**

A 53-year-old woman had undergone left ocular enucleation because of choroidal malignant melanoma 13 years prior to admission. She visited a nearby clinic with complaints of epigastric pain. She was referred to our hospital because a giant liver tumor was observed on abdominal ultrasonography. Enhanced computed tomography revealed multiple liver tumors in the right lobe, 49 mm in diameter with ring enhancement in subsegment (S) 5/6, and 14 and 8 mm without any enhancement in S7 and S5, respectively. On magnetic resonance imaging, the main tumor showed high intensity on T1-weighted with fat suppression, suggesting melanin deposition. Based on the diagnosis of multiple liver metastases of choroidal malignant melanoma, right hepatectomy and regional lymphadenectomy were performed. She was discharged without postoperative complications. Histological examination revealed that all tumors were metastatic malignant melanoma. She was treated with nivolumab postoperatively, and no recurrences were observed during 22 months of follow-up.

**Conclusions:**

Aggressive surgery plus adjuvant nivolumab appears to be a promising treatment for choroidal malignant melanoma with late-onset liver metastases.

## Background

Choroidal malignant melanoma is the most common primary malignant tumor of the eye in adults. It is highly likely that micro-metastases of malignant melanoma originating from the eye via systemic circulation have previously occurred in other organs following the detection of liver metastases of this disease. Therefore, local therapy, including liver resection, is not generally acceptable as a curative therapeutic option [1]. Although patients may benefit from liver resection in selected cases, patients with metastatic melanoma have a median survival of 6 months [2–4].

Currently, immunotherapy is the standard therapy for unresectable or metastatic melanoma, and the prognosis of metastatic melanoma has improved [5]. Therefore, resection of recurrent foci without morphological residual lesion plus adjuvant immunotherapy with immune-checkpoint inhibitor may be beneficial.

This study reports a case of late-onset liver metastases of choroidal malignant melanoma, successfully treated with hepatectomy and postoperative nivolumab.

## Case presentation

A 53-year-old woman underwent left ocular enucleation for choroidal malignant melanoma 13 years prior to admission. Histopathological examination of the enucleated left eye showed a spindle-type choroidal melanoma that did not invade the sclera. Subsequently, the patient was followed up annually by an ophthalmologist (YH).

**Fig. 1:**
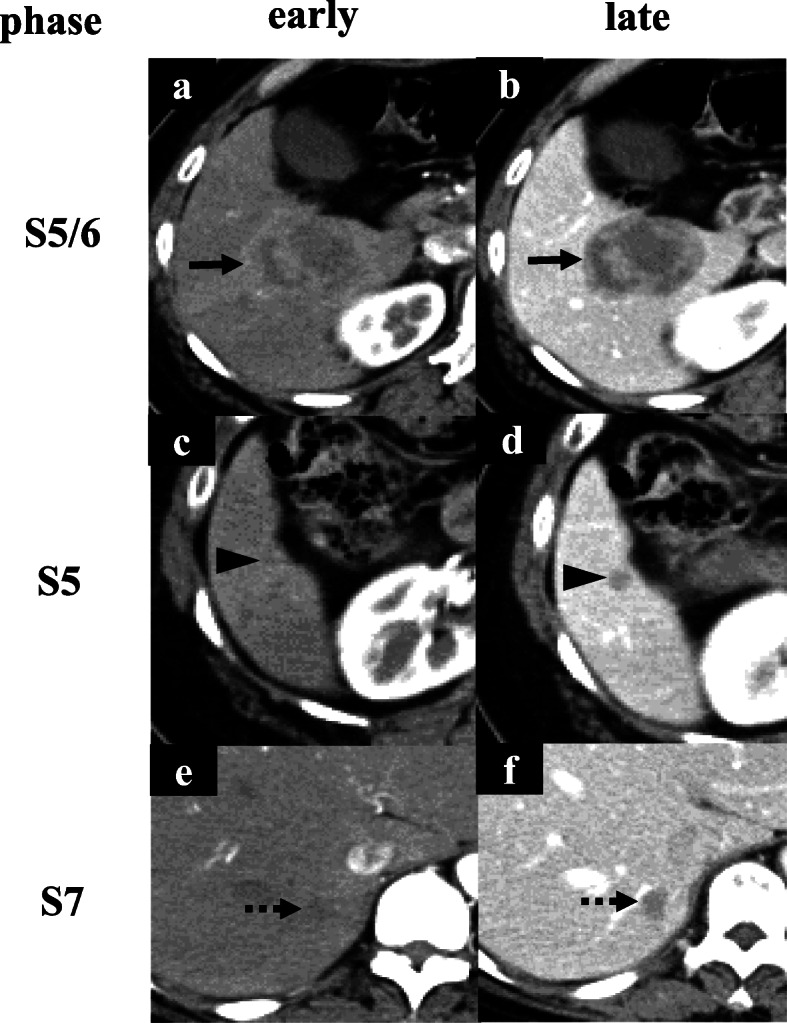
Enhanced CT (computed tomography) findings. **a** Enhanced CT showed a tumor with ring enhancement in S5/6 (black arrow). **c**, **e** S5 and S7 tumors were not observed in the early phase (black arrowhead, dotted black arrow). **b**, **d**, **f** All three tumors were visualized as low-density regions in the late phase

**Fig. 2:**
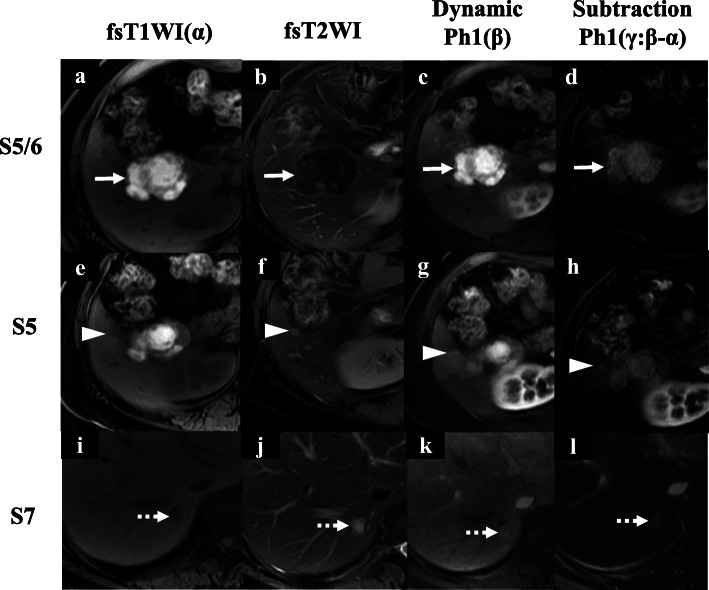
Gadoxetic acid-enhanced MRI (magnetic resonance imaging) findings. **a**–**d** S5/6 tumor showed very high-intensity regions on fat suppression T1-weighted imaging (fsT1WI), low intensity on fsT2WI, high intensity in dynamic Ph1, and strong enhancement in subtraction images (dynamic Ph1 – fsT1WI) (white arrow). **e**–**l** S5 and S7 tumors were visualized as low-intensity regions on fsT1WI, high intensity on fsT2WI, iso-intensity in dynamic Ph1, and weak enhancement in subtraction images (dynamic Ph1 – fsT1WI) (white arrowhead and dotted white arrow)

**Fig. 3:**
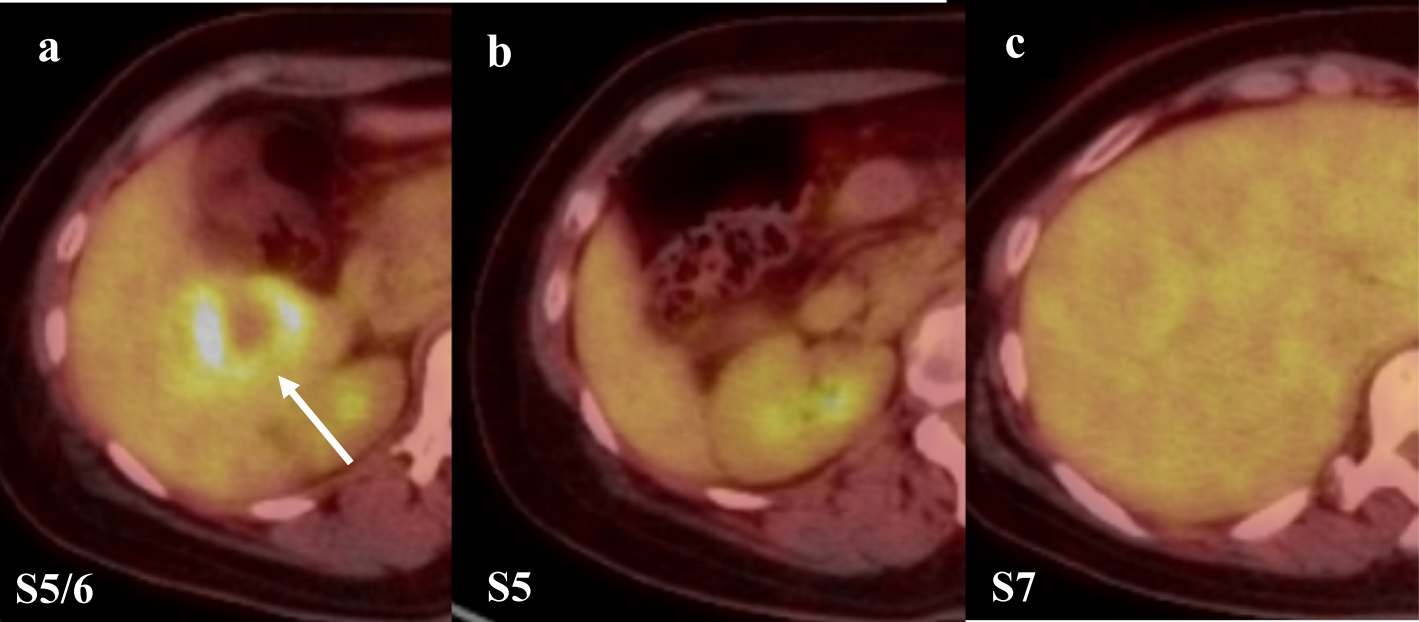
F-18-fluoro-2-deoxyglucose positron emission tomography-computed tomography (FDG PET-CT) findings. **a** FDG PET-CT showed FDG deposition in S5/6: standardized uptake value max 6.9 (white arrow). **b**, **c** FDG PET-CT showed no FDG deposition

She visited a clinic with complaints of epigastric pain. A large liver tumor was observed on abdominal ultrasonography, and she was referred to our hospital. There were no elevations in serum tumor markers (AFP, PIVKA-II, 5-SCD, or NSE), and her liver was in good condition: Child-Pugh score was 5 points, class A. Liver damage was defined as grade A, according to the Liver Cancer Study Group of Japan [6]. Endoscopy showed no gastrointestinal malignant lesions. Enhanced computed tomography (CT) revealed multiple liver tumors in the right lobe, 49 mm in diameter with ring enhancement in subsegment (S) 5/6, and 14 and 8 mm without any enhancement in S7 and S5, respectively (Fig. 1). Lymph node swelling was not observed. Lung CT revealed no definite lung metastasis except for a small nodule, which had already been detected during the primary surgery. On magnetic resonance imaging (MRI), the main lesion showed very high intensity on T1-weighted with fat suppression, suggesting melanin deposition (Fig. 2). The other two lesions indicated low and high intensities on T1-weighted with fat suppression and on T2-weighted imaging, respectively. Subtraction images displayed enhancement in all three tumors. Whole-body F-18-fluoro-2-deoxyglucose (FDG) positron emission tomography-computed tomography (PET-CT) scan revealed FDG deposition in the main tumor, unlike the others (Fig. 3). These tumors were diagnosed as multiple liver metastases of choroidal malignant melanoma.

**Fig. 4:**
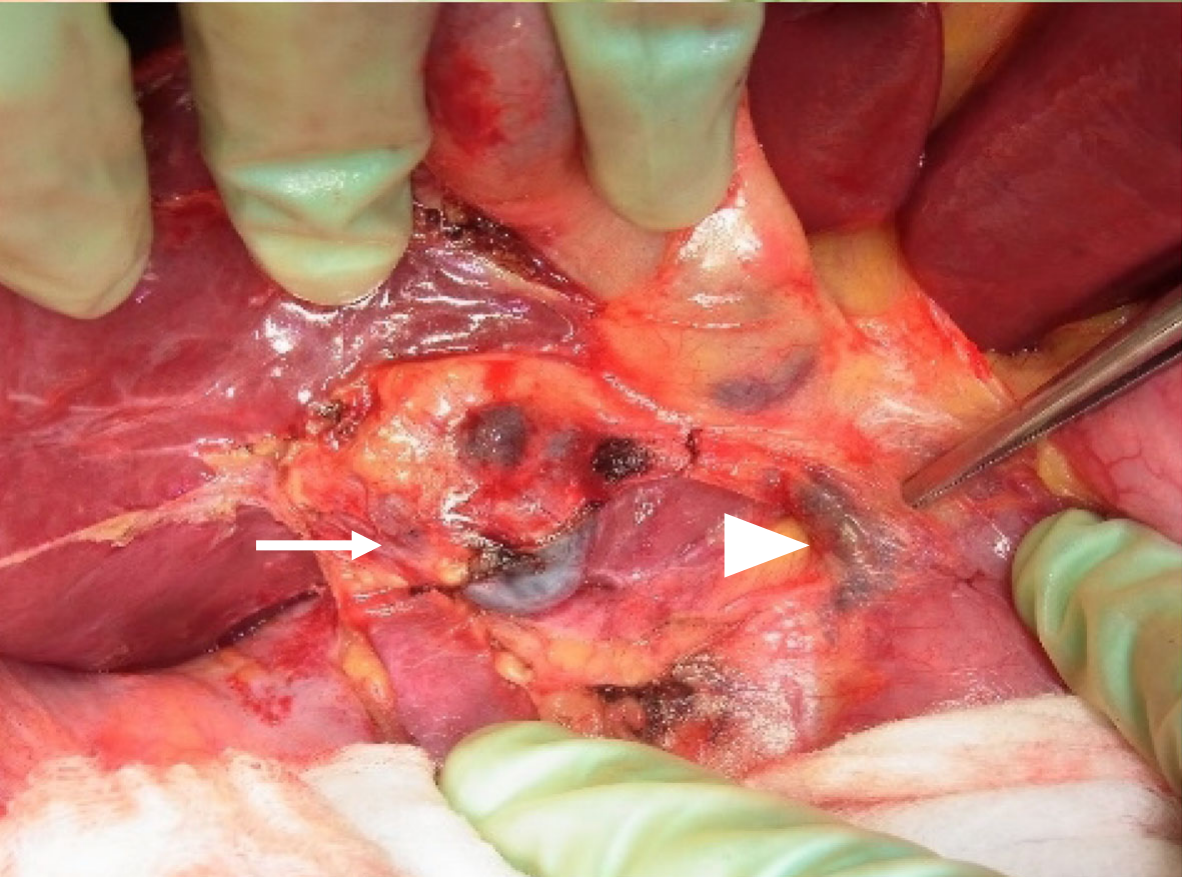
Intraoperative findings. The S5/6 tumor (white arrow) had invaded the liver capsule. Perihilar lymph nodes (white arrowhead) were resected under the suspicion of lymph node metastases

A right hepatectomy was performed. Black swollen lymph nodes in the hepatoduodenal ligament were resected based on the suspicion of lymph node metastases (Fig. 4). The operation time was 262 min, and blood loss was 380 ml. She was discharged without postoperative complications.

**Fig. 5:**
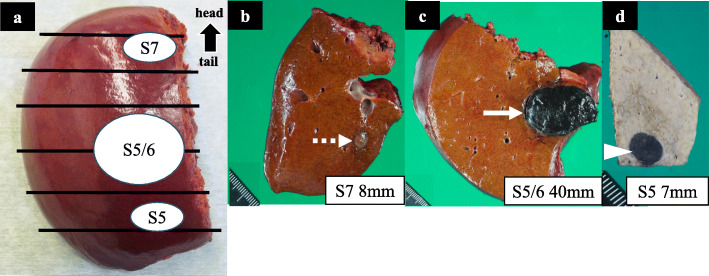
Macroscopic findings. **a** Overview of resected specimen. **b**–**d** The S5/6 (white arrow) and S5 (white arrowhead) tumors were melanotic, whereas the S7 tumor (dotted white arrow) was not

**Fig. 6:**
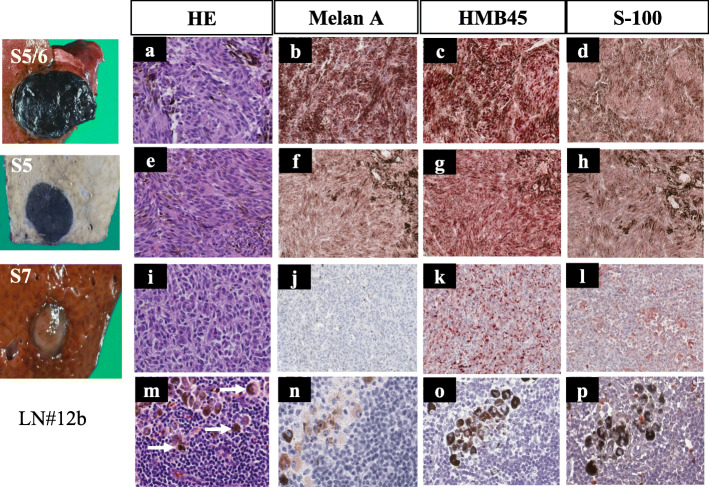
Histopathological examination of the tumors. **a**, **e**, **i** Spindle-shape atypical cells were seen in all three tumors in HE staining. **b**, **c**, **d** The S5/6 tumor showed strong immunostaining intensity for Melan A, HMB45, and S-100, respectively. **f**–**h** S5 tumor showed weak immunostaining intensity for Melan A, HMB45, and S-100, respectively. **j**–**l** S7 tumor showed very weak immunostaining intensity for Melan A, HMB45, and S-100, respectively. **m**–**p** Tumor cells were not observed in the lymph nodes, although the melanin was very rich (white arrow)

On gross inspection, the S5/6 and S5 tumors were observed to be melanotic, unlike the third tumor (Fig. 5). On microscopy, spindle-shape atypical cells were observed in all three tumors. The S5/6 tumor showed strong immunostaining for Melan A, HMB45, and S-100, while the other two tumors showed weak staining (Fig. 6). Programmed death (PD)-ligand 1 expression was not evaluated. Although immunoreactivity was partially different among the three tumors, all tumors were diagnosed as malignant liver metastases with spindle-shape atypical cells and HMB-45-positive cells. Spindle-shape atypical cells were not observed in the perihilar black lymph nodes, although there was a rich melanin deposition. Genetic screening revealed the v-raf murine sarcoma viral oncogene homolog B1 (BRAF) status as wild-type. She was treated with nivolumab (3 mg/kg) every 2 weeks, and she was followed up with whole-body PET-CT and head MRI scans every 6 months according to the National Comprehensive Cancer Network guidelines for cutaneous melanoma [7]. No recurrence was observed for 22 months after surgery. She has not suffered from any serious adverse events.

## Discussion

Choroidal malignant melanoma is the most common primary malignant tumor of the eye in adults, although there are only about 50 cases per year in Japan [8]. Despite local optimal treatment (surgery or radiation) for primary lesion, choroidal malignant melanoma occasionally shows late-onset metastasis: the median disease-free interval is 22.3 years [9]. The prognosis after detection of late-onset recurrence of choroidal malignant melanoma has been dismal. Because of high blood flow to the choroid, the mode of metastatic spread of choroidal melanoma is supposedly hematogenous, with the liver being the most common site [10]. For a long time, liver resection of melanoma had remained controversial. Despite attempts at curative resection, the overwhelming majority (75%) of patients with metastatic melanoma experienced disease recurrence [11]. The median time to recurrence after hepatic resection was 8.3 months. Mariani et al. reported on the surgical management of liver metastases from uveal melanoma [12]; four important survival indicators were described as follows: long disease-free interval (> 24 months), small number of recurrent foci (≤ 4 lesions), comprehensiveness of resected metastases, and the presence or absence of miliary disease. Similarly, Herman et al. suggested the following criteria suitable for resection: disease-free interval > 24 months, absence of extra-hepatic disease, presumed completely resectable lesions, and absence of clinical co-morbidities [2]. This case met all aforementioned criteria. A recent meta-analysis still indicated that radical resection of liver metastases from melanoma improves the overall survival compared with non-operative management or incomplete resection [13]. For this reason, radical liver resection was primarily selected.

Nonetheless, adjuvant chemotherapy has been used to extend survival. Patients receiving some form of systemic therapy had longer median survival than patients who exclusively underwent surgery [11]. Nevertheless, the optimal chemotherapy regimen has not been determined.

Nivolumab, a human IgG4 monoclonal antibody against PD-1, and ipilimumab, a human IgG1 monoclonal antibody against cytotoxic T-lymphocyte antigen 4, are approved in several countries for the treatment of metastatic melanoma [5, 14, 15]. There is an adjuvant treatment strategy in patients with sufficiently high risk of developing metastatic disease. Recently, the Food and Drug Administration approved nivolumab as adjuvant therapy in patients with resected stage IIIB, IIIC, or IV melanoma [5]. Patients who received nivolumab as adjuvant therapy after tumor resection benefitted regardless of the PD-L1 or BRAF status. Under treatment with nivolumab, the estimated 1-year recurrence-free survival in patients with stage IV is 63% [5]. Although the result of overall survival and efficacy for organ metastasis remain unclear, aggressive liver resection can be performed even in the context of multiple liver metastases because there is supporting evidence for the use of adjuvant chemotherapy (nivolumab). Conversion surgery after nivolumab is one of the treatment options to avoid the possibility of an early recurrence. On the contrary, PD-1 antibody is supposedly not effective for choroidal malignant melanoma, unlike cutaneous malignant melanoma [16]. It is reasonable and technically feasible to primarily select surgery in the case of optimal liver function. Combination therapy of nivolumab and ipilimumab for metastatic uveal melanoma appeared to be significantly superior to monotherapy [17]. However, 39.1% of patients treated with combination therapy experienced severe adverse event.

Based on the results of phase 3 trial, it is considered that the adjuvant chemotherapy (nivolumab) may be terminated if there is no recurrence 1 year after surgery [5]. In this case, we similarly planned to terminate nivolumab because no recurrence was observed 1 year after liver resection. However, a preoperative CT scan revealed a small nodule in the lung. This nodule had already been detected at the time of the primary surgery (13 years before) and was not changed when liver metastases were detected. Prior to the hepatectomy, we consulted with a respiratory surgeon and opted for observation unless the pulmonary nodule would become enlarged. The pulmonary nodule has been unchanged until the most recent PET-CT scan. Although we continue nivolumab following the patient’s explicit request, no remarkable changes were observed 22 months after the liver resection.

Patients with amelanotic metastatic melanomas had longer survival time than those with pigmented tumors [18]. Tumor appearance depends on the melanin volume, and the pigmentation level reflects malignant potential. In this case, macroscopic examination indicated that two tumors in the anterior section were melanotic, unlike the third tumor, which was not. We could not find any reports supporting this unique event. Notably, immunostaining for HMB45 is useful in differentiating malignant melanoma from benign, even if lesions are amelanotic [19].

Importantly, in radiologic imaging, tumor appearance can similarly depend on the melanin volume. Gadoxetic acid (Gd-EOB)-enhanced MRI is more useful than multidetector CT for the detection of multiple metastatic foci [20]. It can provide information not only about the vascularization of the lesions in different phases of contrast circulation but also the functional parameters in the delayed hepatobiliary phase. However, it is difficult to value the vascularity because of high-signal intensity in pre-contrast T1-weighted imaging [21]. In this regard, subtraction technique should be used to evaluate enhancement.

In this case, perihilar black lymph nodes were resected under the suspicion of lymph node metastases; however, these nodes pathologically showed no metastatic melanoma cells. Assumingly, either the perihilar lymph nodes took up the melanin released from hepatic metastases or the metastatic melanoma cells were degraded by immunocytes, and only melanin could be deposited in the lymph nodes; however, the evidence is lacking.

## Conclusions

Multimodal therapy with surgery and adjuvant nivolumab has the potential of improving the prognosis of late-onset liver metastasis from choroidal melanoma.

## Abbreviations

CT: Computed tomography
S: Subsegment
MRI: Magnetic resonance imaging
FDG: F-18-fluoro-2-deoxyglucose
PET-CT: Positron emission tomography-computed tomography
PD: Programmed death
BRAF: V-raf murine sarcoma viral oncogene homolog B1
